# Sex Differences in Clinical and Patient-Reported Outcomes in Transcatheter Aortic Valve Implantation: The TAVI-COMIC Trial

**DOI:** 10.1016/j.jacadv.2025.102012

**Published:** 2025-07-18

**Authors:** Tharusan Thevathasan, Fabian Reitz, Constantin Hornig, David M. Leistner, Henryk Dreger, Simon Sündermann, Isabel Mattig, Axel Unbehaun, Arash Haghikia, Carsten Skurk, Hristian Hinkov, Berit Zirkelbach, Karl Stangl, Volkmar Falk, Ulf Landmesser, Gerhard Hindricks, Verena Stangl, Anna Brand

**Affiliations:** a Department of Cardiology, Angiology and Intensive Care Medicine, Deutsches Herzzentrum der Charité (DHZC), Campus Benjamin Franklin, Berlin, Germany; b Berlin Institute of Health, Berlin, Germany; c DZHK (German Centre for Cardiovascular Research), Berlin, Germany; d Department of Cardiology, Angiology and Intensive Care Medicine, Deutsches Herzzentrum der Charité (DHZC), Campus Mitte, Berlin, Germany; e Medizinische Klinik 3, Kardiologie und Angiologie, Universitätsmedizin Frankfurt, Cardio-Pulmonary Institute (CPI), Frankfurt am Main, Germany; f Department of Cardiology, Angiology and Intensive Care Medicine, Deutsches Herzzentrum der Charité (DHZC), Campus Virchow Klinikum, Berlin, Germany; g Department of Cardiothoracic and Vascular Surgery, Deutsches Herzzentrum der Charité (DHZC), Campus Virchow Klinikum, Berlin, Germany; h Department of Cardiology, University Hospital St Josef-Hospital Bochum, Bochum, Germany; i Clinic for Internal Medicine-Cardiology and Conservative Intensive Care Medicine, Vivantes Humboldt-Klinikum, Berlin, Germany

**Keywords:** patient-reported outcomes, sex difference, transcatheter aortic valve implantation

## Abstract

**Background:**

Transcatheter aortic valve implantation (TAVI) is an established treatment for severe aortic valve stenosis. Data regarding sex-specific differences in patient-centered endpoints, such as patient-reported outcomes (PROs), and in sociodemographic characteristics are scarce.

**Objectives:**

This study aimed to investigate patient-centered, sex-specific characteristics and their associations with the overall patient outcome, that is, clinical course and PROs in patients undergoing TAVI.

**Methods:**

This analysis of the TAVI-COMIC randomized controlled trial included 299 patients undergoing TAVI at 2 tertiary care centers. Data were collected for sociodemographic characteristics, PROs, and postprocedural outcomes for up to 90 days.

**Results:**

Male patients undergoing TAVI had significantly higher educational attainment with more university degrees (34.2% vs 13.0%, *P* < 0.001). Female patients were significantly less often in a relationship (29.7% vs 73.3%, *P* < 0.001). Females reported significantly higher anxiety levels before TAVI (State-Trait Anxiety Inventory: 42 vs 39 points, *P* < 0.001). Postprocedure, female patients experienced longer hospital stays (8 vs 7 days) and reported greater difficulties with daily activities such as walking (41.7% vs 29.8%), shopping (41.7% vs 27.2%), helplessness (35.7% vs 10.5%), and depression (45.2% vs 25.4%). Female patients were more frequently discharged to a nursing facility (11.6% vs 3.7%).

**Conclusions:**

This study highlights significant sex-specific differences in educational and relationship status, anxiety levels, as well as length of hospitalization and PROs following TAVI. Female patients showed higher anxiety and more frequent postprocedural challenges. These findings underscore the need for tailored pre- and post-procedural care to address the distinct needs of male and female TAVI patients.

Transcatheter aortic valve implantation (TAVI) has become an established treatment option for patients with severe aortic valve stenosis across all risk categories for surgical aortic valve replacement. However, the cohort of patients undergoing TAVI for severe aortic valve stenosis still predominantly consists of elderly individuals, mainly octogenarians, with numerous comorbidities.^1^ Notably, the incidence of post-TAVI complications varies by sex, with women having a higher risk of bleeding, vascular complications, and strokes.^2^, ^3^, ^4^, ^5^

For this patient population, alleviating symptoms, enhancing functional status, and improving quality of life are crucial, if not more important, than mere survival status and complications. Over the past decade, there has been a significant shift toward assessing medical interventions through both clinical and nonclinical parameters that reflect improvements in daily life and are of greater importance for patients and their families. Patient-reported outcomes (PROs) are standardized and validated tools to assess patients' self-reported health status. PROs aim to capture various dimensions that matter most to patients prior to, during, and after medical interventions, including physical, psychological, and social aspects. Although the body of data on PROs in TAVI patients is expanding, it has remained inadequate for thoroughly analyzing sex differences and possible associations with sex-specific sociodemographic factors.

This gap highlights the necessity for more comprehensive studies that specifically examine how male and female patients experience and report outcomes differently before, during, and after TAVI. Addressing these differences could potentially improve personalized care strategies and optimize overall treatment efficacy for both sexes. Therefore, the aim of this study was to analyze sex-specific differences in clinical and nonclinical outcomes of male and female patients undergoing TAVI.

## Methods

### Study setting and population

This study was conducted at 2 independent tertiary care centers at Deutsches Herzzentrum der Charité in Berlin, Germany. Both tertiary care centers are high-volume, university-affiliated heart centers, performing approximately 1,300 TAVI procedures per year, with procedures conducted by experienced interventional cardiologists certified for structural heart interventions. This study is an analysis of the preregistered randomized controlled trial “TAVI-COMIC” (protocol: DRKS00021661)^6^^,^^7^ and received approval from the Institutional Review Board (registration numbers EA1/139/20 and EA1/171/24, Ethikkommission Charité – Universitätsmedizin Berlin). The study adheres to the Strengthening the Reporting of Observational Studies in Epidemiology guidelines, and the work has been carried out in accordance with “The Code of Ethics of the World Medical Association” (Declaration of Helsinki). The study was supported by the DGK Center for Health Services Research (DGK Zentrum für kardiologische Versorgungsforschung, DGK-ZfKVF). The study sponsor did not participate in the study procedure and was neither involved in the collection, analysis, and interpretation of data; in writing of the report; nor in the decision to submit the paper for publication.

In the TAVI-COMIC trial, 301 adult male and female patients were randomized to receive either a standardized consent process (reference group) or consent including a brochure containing medical graphics (“comics”; interventional group) prior to TAVI. The original trial focused on the impact of visual aids on patient comprehension and anxiety, while the current secondary analysis investigated sex differences in clinical and PROs. The results of the primary trial demonstrated that employing medical graphics to educate patients about TAVI significantly enhanced patient comprehension and diminished periprocedural anxiety. In this analysis, patients who underwent TAVI were assessed for clinical and patient-reported differences between both sexes before, during, and until 90 (Q1-Q3: 89 to 94) days after the procedure.

### Data collection and sources

Data were primarily collected as part of the TAVI-COMIC trial: Demographic data included age, sex, body mass index (BMI), educational attainment (school and university degrees as well as job training), mother tongue, and relationship status. Educational attainment and relationship status were used as proxies for socioeconomic status. Relationship status refers to whether the patient was single, in a registered partnership (eg, married or cohabiting), or widowed.

Pre- and intra-procedural parameters included the Essential Frailty Toolset (EFT), Montreal Cognitive Assessment (MoCA), periprocedural anxiety levels measured by the State-Trait Anxiety Inventory (STAI), patient satisfaction assessed by the validated Client Satisfaction Questionnaire (CSQ-8), and patient understanding using a specific questionnaire, as previously published.^6^ The EFT was employed to assess frailty as a proxy for the patient’s baseline health status prior to TAVI, with a score >2 indicating frailty.^8^ Patients with a MoCA score below 26 points were classified as having mild cognitive disorder. Conversely, a higher cumulative score on the CSQ-8 was indicative of greater patient satisfaction. State anxiety was assessed using a 20-item questionnaire based on a 4-point Likert scale, yielding a total score range between 20 and 80 points, with higher scores indicating greater levels of anxiety. In working adult populations, reference values for state anxiety have been reported as 35.72 (±10.40) for men and 35.20 (±10.61) for women.^9^ To assess whether patients comprehended the consenting process for the planned TAVI procedure, a structured questionnaire consisting of 14 items evaluating key aspects of the intervention, risks, and alternatives was provided. Answers were scored as “correct” or “incorrect,” resulting in a sum range of 0 to 14 points. Additionally, laboratory values (such as hemoglobin and renal function), echocardiographic parameters (including left ventricular ejection fraction as well as mean and maximum aortic valve pressure gradients), computer tomographic parameters (including aortic annular perimeter and area), the type of aortic valve prosthesis (self-expandable vs balloon-expandable), and duration of anesthesia were collected.

Postprocedural parameters included echocardiographic measures after 1 day (such as mean and maximum transaortic valve pressure gradients), medical complications during the index hospitalization after TAVI (such as bleeding, cardiac arrhythmias, cardiac conduction disorders, pneumonia, and stroke), length of stay (LOS) in the TAVI unit and the hospital, as well as 90-day PROs, including overall health status, walking ability, daily activities (such as shopping), and symptoms (such as dizziness, syncope, dyspnea, angina, helplessness, and depression). Patients were invited by letter to fill in a questionnaire assessing the aforementioned PROs, as previously published.^6^

Laboratory and complication data were collected from electronic patient information systems, including COPRA System GmbH, Centricity RIS-i (GE Healthcare), and SAP ishmed hospital information system, as described in a previous publication.^10^ All retrieved data were manually controlled by 2 independent investigators through careful review of discharge reports and notes at each hospital site.

### Outcomes

The primary outcomes of this study were defined as sex-specific differences in patient characteristics and patient-reported evaluation measures, such as periprocedural levels of anxiety, satisfaction, and comprehension, as well as postprocedural parameters, which included medical complications, LOS in the TAVI unit and hospital, and 90-day PROs.

### Data analysis

Data analysis was performed using R Core Team 2020. Normality was assessed using Shapiro-Wilk analysis. Categorical and continuous variables were compared using chi-square tests and Student’s *t*-tests (normally distributed) or Fisher exact test and Wilcoxon-Mann-Whitney U-test (non-normally distributed), respectively. Normally distributed continuous variables are expressed as mean (SD), non-normally distributed variables as median (IQR), and categorical variables as frequency (percentage).

Univariable and multivariable logistic as well as negative binomial regression analyses were conducted to evaluate the association between patient sex and outcomes. The regression models were adjusted for relevant covariates, including age, BMI, and CSQ-8. Logistic regression was applied for binary outcomes (eg, depressive symptoms or helplessness), while negative binomial regression was used for count-based outcomes with overdispersion (eg, hospital LOS). Model selection was informed by likelihood ratio testing. No offset term was used, as all patients had similar follow-up duration. Results are presented as adjusted ORs or adjusted incidence rate ratios with 95% CIs and *P* values. A 2-tailed *P* value of <0.05 was considered statistically significant.

With an exploratory intent, the interaction effect of graphic-based consenting on the association between patient sex and primary outcomes was analyzed. Additionally, the association between sociodemographic factors and PROs was investigated using multivariable logistic regression, adjusted for age, BMI, and CSQ-8 scores.

## Results

**Figure 1:**
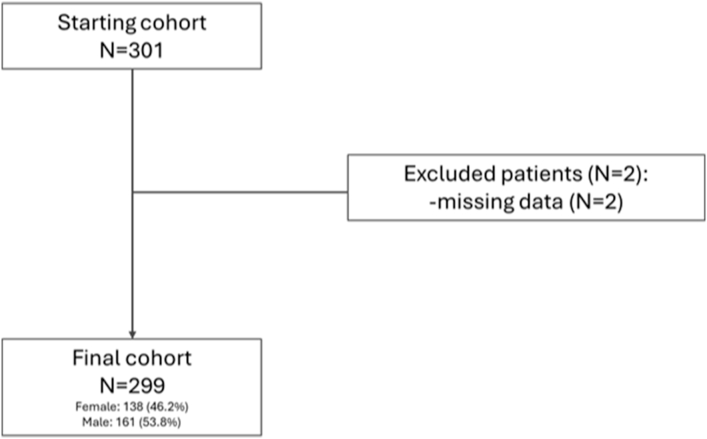
**Patient Flowchart** The figure illustrates the study flowchart for the analysis of the TAVI-COMIC randomized controlled trial. Of the 301 patients initially enrolled, 299 were included in the final analysis. Among these, 161 (53.8%) were male and 138 (46.2%) were female. Two patients were excluded due to incomplete data.

Out of a total of 301 patients, 299 patients were included in this study, of which 161 of 299 (53.8%) were males and 138 of 299 (46.2%) were females. Two patients were excluded due to missing data (Figure 1). The average age was 81 (±7) years, and the average BMI was 26 (Q1-Q3: 23-29) kg/m^2^. Notably, male patients undergoing TAVI possessed over twice the number of high school degrees (56/161 [34.8%] vs 23/138 [16.7%], *P* < 0.001) and university degrees (55/161 [34.2%] vs 18/138 [13.0%], *P* < 0.001) as female patients. Furthermore, female patients were more often single (32/138 [23.2%] compared to 24/161 [14.9%]) and widowed (65/138 [47.1%] compared to 19/161 [11.8%]) and less frequently in a registered partnership (41/138 [29.7%] compared to 118/161 [73.3%]) (each *P* < 0.001) (Central Illustration). Based on patients’ postal codes, 271 of 299 [90.6%] resided in urban areas. There was no statistically significant difference in residential setting between male and female patients (urban: 143/161 males [88.8%] vs 128/138 females [92.7%], *P* = 0.345) (Table 1). Educational attainment also did not differ significantly between urban and rural residents: 69 of 271 (25.5%) patients with university degrees from urban regions vs 9 of 28 (32.1%) from rural regions (*P* = 0.95).

**Central Illustration:**
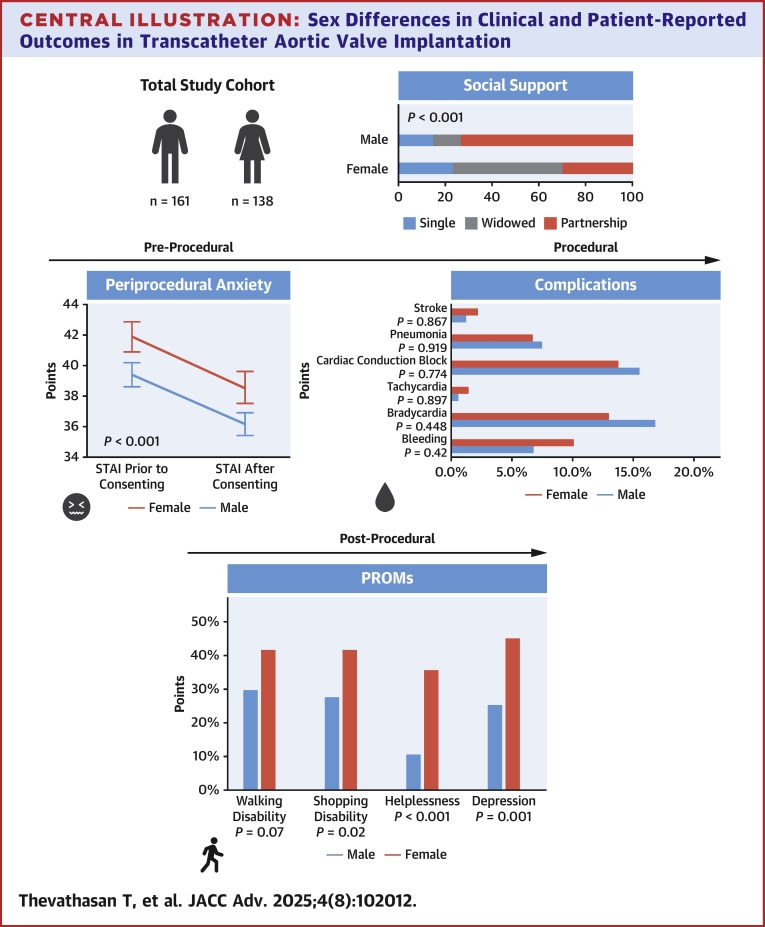
**Sex Differences in Clinical and Patient-Reported Outcomes in Transcatheter Aortic Valve Implantation** The Central Illustration summarizes the key findings from the analysis of the TAVI-COMIC randomized controlled trial. Among the 299 included patients, 54% were male and 46% were female. The proportion of single or widowed female patients was significantly higher compared to male patients, and female patients were less likely to have completed high school or university education. Female patients exhibited higher levels of preprocedural anxiety, as measured by the STAI. During the TAVI procedure, balloon-expandable valves were less frequently utilized in female patients. Medical complications following TAVI were comparable between both sexes. However, with respect to patient-reported outcomes up to 90 days following TAVI, female patients more frequently reported difficulties, including walking and shopping disabilities, as well as feelings of helplessness and depression. PROMS = patient reported outcome measures; STAI = State-Trait Anxiety Inventory; TAVI = transcatheter aortic valve implantation.

**Table 1:** Characteristics of Male and Female Patients Undergoing TAVI

	Male Patients (n = 161)	Female Patients (n = 138)	Total (N = 299)	*P* Value
Patient demographics				
Age (y), mean (SD)	80.2 (7.47)	82.0 (5.59)	81.0 (6.72)	0.05
Body mass index (kg/m^2^), median [IQR]	25.9 [23.4-28.4]	25.4 [21.8-29.8]	25.7 [22.7-29.1]	0.59
High school degree, n/N (%)	56/161 (34.8%)	23/138 (16.7%)	79/299 (26.4%)	<0.001
University degree, n/N (%)	55/161 (34.2%)	18/138 (13.0%)	73/299 (24.4%)	<0.001
Completed job training, n/N (%)	98/161 (60.2%)	104/138 (75%)	202/299 (67.6%)	<0.001
Jobless or retired, n/N (%)	154/161 (95.7%)	137/138 (99.3%)	291/299 (97.3%)	0.12
German mother tongue, n/N (%)	156/161 (96.9%)	136/138 (98.6%)	292/299 (97.7%)	0.58
Living in an urban area, n/N (%)	143/161 (88.8%)	128/138 (92.7%)	271/299 (90.6%)	0.35
Relationship status				
Single, n/N (%)	24/161 (14.9%)	32/138 (23.2%)	56/299 (18.7%)	<0.001
Widowed, n/N (%)	19/161 (11.8%)	65/138 (47.1%)	84/299 (28.1%)	<0.001
Registered partnership, n/N (%)	118/161 (73.3%)	41/138 (29.7%)	159/299 (53.2%)	<0.001
Preprocedural characteristics				
Essential Frailty Toolset (>2 points)	22/161 (13.7%)	17/138 (12.3%)	39/299 (13.0%)	0.86
Mild cognitive impairment (MoCA <26)	83/161 (51.6%)	80/138 (58.0%)	163/299 (54.5%)	0.32
STAI prior to consenting, median [IQR]	39.0 [36.0-43.0]	42.0 [38.0-46.0]	40.0 [37.0-44.0]	<0.001
STAI after consenting, median [IQR]	35.0 [32.0-39.0]	38.0 [34.0-43.0]	36.0 [32.0-41.0]	<0.001
Sum of CSQ-8, median [IQR]	26.0 [25.0-29.0]	27.5 [25.0-29.0]	27.0 [25.0-29.0]	0.53
Consenting was considered to be relaxing prior to procedure (n [%])	74/161 (46.0%)	34/138 (24.6%)	108/299 (36.1%)	<0.001
Sum of comprehension score, median [IQR]	13.0 [11.0-13.0]	12.0 [11.0-13.0]	12.0 [11.0-13.0]	0.08
Did not understand conventional consenting prior to TAVI, n/N (%)	87/161 (54.0%)	104/138 (75.4%)	191/299 (63.9%)	<0.01
Laboratory parameters				
Hemoglobin (g/dL), median [IQR]	12.3 [10.7-13.9]	11.8 [11.0-13.1]	12.1 [10.8-13.4]	0.07
Creatinine (mg/dL), median [IQR]	1.41 [0.99-1.49]	1.08 [0.84-1.19]	1.26 [0.89-1.33]	<0.001
Glomerular filtration rate (ml/min/1.73 m^2^), mean (SD)	57.5 (19.8)	56.6 (17.5)	57.1 (18.8)	0.51
Echocardiographic parameters				
LVEF (%), median [IQR]	51.6 [45.0-60.0]	57.6 [54.0-64.0]	54.4 [50.0-60.0]	<0.001
Maximum transaortic valve pressure gradient (mm Hg), mean (SD)	68.0 (20.4)	67.4 (16.9)	67.7 (18.9)	0.64
Mean transaortic valve pressure gradient (mm Hg), mean (SD)	42.7 (13.6)	41.9 (11.5)	42.3 (12.7)	0.36
Computer tomographic parameters				
Aortic annular perimeter (mm)	82 (6)	73 (6)	78 (8)	<0.001
Aortic annular area (mm^2^)	522 (80)	408 (77)	468 (97)	<0.001
Intraprocedural characteristics				
Type of valve prosthesis				
Self-expandable, n/N (%)	50/161 (31.1%)	96/138 (69.6%)	146/299 (48.8%)	<0.001
Balloon-expandable, n/N (%)	110/161 (68.3%)	42/138 (30.4%)	152/299 (50.8%)	<0.001
Duration of anesthesia (min), median [IQR]	67.0 [56.0-84.0]	67.0 [56.0-83.0]	67.0 [56.0-84.0]	0.85
Postprocedural characteristics				
Echocardiographic parameters				
Maximum transaortic valve pressure gradient (mm Hg), mean (SD)	19.4 (8.02)	19.2 (8.36)	19.3 (8.16)	0.77
Mean transaortic valve pressure gradient (mm Hg), mean (SD)	10.6 (4.59)	10.4 (4.93)	10.5 (4.74)	0.54
Medical complications during hospital stay after TAVI				
Bleeding, n/N [95% CI]	11/161 [95% CI: 2.9%-10.8%]	14/138 [95% CI: 5%-15.2%]	25/299 [95% CI: 5.2%-11.6%]	0.42
Bradycardia, n/N [95% CI]	27/161 [95% CI: 11%-22.7%]	18/138 [95% CI: 7.4%-18.7%]	45/299 [95% CI: 11%-19.2%]	0.45
Tachycardia, n/N [95% CI]	1/161 [95% CI: 0%-1.9%]	2/138 [95% CI: 0%-3.5%]	3/299 [95% CI: 0%-2.2%]	0.90
Cardiac conduction block, n/N [95% CI]	25/161 [95% CI: 9.9%-21.3%]	19/138 [95% CI: 8%-20%]	44/299 [95% CI: 10.7%-18.8%]	0.77
Pneumonia, n/N [95% CI]	12/161 [95% CI: 3.4%-12%]	9/138 [95% CI: 2.4%-10.7%]	21/299 [95% CI: 4.1%-10%]	0.92
Stroke, n/N [95% CI]	2/161 [95% CI: 0%-3%]	3/138 [95% CI: 0%-5%]	5/299 [95% CI: 0.2%-3.1%]	0.87
Hospital data				
TAVI unit length of stay (d), median [IQR]	4.0 [3.0-4.0]	4.0 [3.0-5.0]	4.0 [3.0-5.0]	0.02
Hospital length of stay (d), median [IQR]	7.0 [6.0-8.0]	8.0 [6.0-11.0]	7.0 [6.0-9.0]	<0.001
Discharge to home, n/N (%)	145/161 (90.1%)	105/138 (76.1%)	250/299 (83.6%)	0.01
Discharge to nursing facility, n/N (%)	6/161 (3.7%)	16/138 (11.6%)	22/299 (7.4%)	
Referral to another hospital or department, n/N (%)	7/161 (4.3%)	14/138 (10.1%)	21/299 (7.0%)	
Patient-reported outcomes at 90 d after TAVI				
Good or excellent general health status, n/N (%)	95/114 (83.3%)	65/84 (77.4%)	160/198 (80.8%)	0.48
General health status improved after TAVI, n/N (%)	84/114 (73.7%)	57/84 (67.9%)	141/198 (71.2%)	0.54
15-min walking disability, n/N (%)	34/114 (29.8%)	35/84 (41.7%)	69/198 (34.8%)	0.07
Helplessness, n/N (%)	12/114 (10.5%)	30/84 (35.7%)	42/198 (21.2%)	<0.001
Depressive symptoms, n/N (%)	29/114 (25.4%)	38/84 (45.2%)	67/198 (33.8%)	0.01
Disability to go shopping, n/N (%)	31/114 (27.2%)	35/84 (41.7%)	66/198 (33.3%)	0.03
Dizziness, n/N (%)	50/114 (43.9%)	40/84 (47.6%)	90/198 (45.5%)	0.65
Syncope, n/N (%)	6/114 (5.3%)	3/84 (3.6%)	9/198 (4.5%)	0.84
Dyspnea (NYHA functional class II-IV), n/N (%)	53/114 (46.5%)	44/84 (52.4%)	97/198 (49.0%)	0.40
Angina (CCS class I-IV), n/N (%)	22/114 (19.3%)	17/84 (20.2%)	39/198 (19.7%)	0.81

Values are n/N (%), mean (SD), or median (IQR). For postprocedural complications, 95% CIs are displayed.

CCS = Canadian Cardiovascular Society; CSQ-8 = Client Satisfaction Questionnaire; LVEF = left ventricular ejection fraction; MoCA = Montreal Cognitive Assessment; STAI = State-Trait Anxiety Inventory; TAVI = transcatheter aortic valve implantation.

### Pre- and intra-procedural sex differences

The proportion of patients classified as frail according to the EFT was comparable between both sexes (male: 22/161 [13.7%] vs female: 17/138 [12.3%], *P* = 0.86), suggesting that both groups entered the procedure with a similar baseline health status. Female patients demonstrated a higher, though not statistically significant, prevalence of mild cognitive disorder as measured by the MoCA (80/138 [58.0%] vs 83/161 [51.6%], *P* = 0.32). They also exhibited significantly higher anxiety levels both preconsent (STAI: 42 [Q1-Q3: 38-46] vs 39 [Q1-Q3: 36-43], *P* < 0.001) and postconsent (STAI: 38 [Q1-Q3: 34-43] vs 35 [Q1-Q3: 32-39] points, *P* < 0.001) compared to their male counterparts. Patient satisfaction, assessed via the CSQ-8, was comparable between both sexes, with females scoring 28 (Q1-Q3: 25-29) points and males scoring 26 (Q1-Q3: 25-29) points (*P* = 0.53). Remarkably, only 34 of 138 (24.6%) female patients found the consenting process beneficial in alleviating preprocedural anxiety compared to 74 of 161 (46.0%) male patients (*P* < 0.001). Both sexes showed similar understanding of the TAVI procedures, with females scoring 12 (Q1-Q3: 11-13) points and males scoring 13 (Q1-Q3: 11-13) points (*P* = 0.08). However, a greater proportion of female patients failed to comprehend the information provided through standard consenting methods without the aid of medical illustrations (104/138 [75.4%] vs 87/161 [54.0%], *P* < 0.01). Additionally, female patients had lower hemoglobin (11.8 [Q1-Q3: 11.0-13.1] vs 12.3 [Q1-Q3: 10.7-13.9] mg/dL, *P* = 0.07) and creatinine levels (1.08 [Q1-Q3: 0.84-1.19] vs 1.41 [Q1-Q3: 0.99-1.49] mg/dL, *P* < 0.001) compared to male patients. Left ventricular ejection fraction was significantly higher in female patients (58% [Q1-Q3: 54-64]) than in male patients (52% [Q1-Q3: 45-60]) (*P* < 0.001). The mean aortic annular perimeter and area were significantly smaller in female patients (*P* < 0.001). Significant differences were accordingly observed in the types of TAVI prostheses utilized (*P* < 0.001): female patients more frequently received self-expandable prostheses (96/138 [69.6%] vs 50/161 [31.1%]), whereas male patients more commonly received balloon-expandable prostheses (110/161 [68.3%] vs 42/138 [30.4%]). Despite the anatomical differences mentioned (smaller aortic annuli), transvalvular pressure gradients before and after TAVI were similar in both sexes (Table 1).

### Postprocedural sex differences

The rates of all postprocedural complications were similar between both sexes. Female patients showed a numerically higher incidence of bleeding (14/138 [95% CI: 5% to 15.2%]) vs 11/161 [95 CI: 2.9% to 10.8%]), although this difference was not statistically significant (Table 1, Table 2). Female patients also had a significantly longer hospital LOS: 8 days (Q1-Q3: 6-11) vs 7 days (Q1-Q3: 6-8). These findings remained significant after adjusting for multiple confounders: incidence rate ratio: 1.14 (95% CI: 1.03-1.27), *P* = 0.01. Interestingly, female patients were more frequently discharged to a nursing facility (16/138 [11.6%] vs 6/161 [3.7%]) or transferred to another department (14/138 [10.1%] vs 7/161 [4.3%]), whereas male patients were predominantly discharged directly to their homes (145/161 [90.1%] vs 105/138 [76.1%], *P* = 0.01).

**Table 2:** Postprocedural Outcomes Following TAVI in Female Patients Compared to Male Patients

	OR or Incidence Rate Ratio (95% CI) Female vs Male	*P* Value	*P* Value for Interaction of Graphic-Based Consent
Complications during the hospital stay after TAVI			
Bleeding	1.57 (0.67-3.72)	0.298	0.47
Bradycardia	0.73 (0.37-1.38)	0.335	0.27
Tachycardia	2.33 (0.21-51.49)	0.497	0.69
Cardiac conduction block	0.86 (0.44-1.67)	0.656	0.64
Pneumonia	0.92 (0.36-2.30)	0.853	0.31
Stroke	1.93 (0.31-15.37)	0.483	0.73
Hospital data			
TAVI unit length of stay	1.08 (0.96-1.21)	0.196	0.63
Hospital length of stay	1.14 (1.03-1.27)	**0.013**	0.68
Patient-reported outcomes at 90 d after TAVI			
Bad health status	0.64 (0.30-1.36)	0.245	0.59
General health status did not improve due to TAVI	0.65 (0.34-1.26)	0.205	0.72
15-min walking disability	1.91 (1.03-3.55)	**0.040**	0.76
Helplessness	5.50 (2.54-12.68)	**<0.001**	0.64
Depressive symptoms	2.62 (1.40-4.98)	**0.003**	0.39
Disability to go shopping	2.35 (1.25-4.47)	**0.009**	0.56
Dizziness	1.28 (0.70-2.33)	0.427	0.71
Syncope	0.70 (0.14-2.87)	0.633	0.15
Dyspnea (NYHA functional class II-IV)	1.42 (0.77-2.64)	0.257	0.78
Angina (CCS class I-IV)	1.08 (0.51-2.24)	0.840	0.08

The risk of each outcome was compared between female and male patients. Analyses were adjusted for age, body mass index, and Client Satisfaction Questionnaire-8. The interaction term was constructed based on patient sex and whether the consent included medical graphics. The *P* value for interaction indicates the effect of this interaction term on the relationship between patient sex and outcomes. A *P*-value <0.05 is indicated in bold.

Abbreviations as in Table 1.

The general health status was comparable between both sexes, with 65 of 84 (77.4%) females and 95 of 114 (83.3%) males reporting good to excellent health after TAVI (*P* = 0.48). However, female patients more frequently experienced disabilities in walking (35/84 [41.7%] vs 34/114 [29.8%], *P* = 0.07) and shopping (35/84 [41.7%] vs 31/114 [27.2%], *P* = 0.03), as well as higher rates of helplessness (30/84 [35.7%] vs 12/114 [10.5%], *P* < 0.001) and depressive symptoms (38/84 [45.2%] vs 29/114 [25.4%], *P* = 0.01) after TAVI. Other symptoms, such as dyspnea, angina, dizziness, or syncope, were similar between both sexes (Table 1). With multivariable adjustment, impairment in shopping (OR: 2.35; 95% CI: 1.25-4.47; *P* = 0.01), disabled walking ability (OR: 1.91; 95% CI: 1.03-3.55; *P* = 0.04), feelings of helplessness (OR: 5.50; 95% CI: 2.54-12.68; *P* < 0.001), and depressive symptoms (OR: 2.62; 95% CI: 1.40-4.98; *P* < 0.01) were higher in female compared to male patients (Table 2). Of note, the intervention of graphic-based consenting did not affect the association between patient sex and outcomes (each P for interaction = n.s.) (Table 2). Additional subanalyses indicated that PROs remained consistent between male and female patients among groups with the same educational level (Supplemental Table 1).

### Exploratory analysis

Exploratory analyses indicated that increased BMI was associated with poorer general health status following TAVI. Additionally, individuals who were single reported higher levels of depressive symptoms after TAVI, while periprocedural anxiety was found to be associated with prolonged hospitalization, feelings of helplessness, depressive symptoms, dyspnea, and angina (Supplemental Table 2).

## Discussion

This analysis of the TAVI-COMIC randomized controlled trial demonstrated significant sex-specific differences in patient-specific characteristics as well as clinical outcomes and PROs following TAVI. Female patients exhibited distinct demographic, procedural, and postprocedural characteristics compared to their male counterparts underscoring the necessity for tailored approaches in the management and care strategies of TAVI patients.

### Preprocedural differences

Female patients in this study had lower educational attainment levels, with fewer high school and university degrees compared to male patients. This disparity in educational background could explain why fewer women understood the TAVI consent procedure, which might have limited their abilities for engagement in the decision-making process. Of note, while the residential setting can affect health literacy and access to care,^11^ this study revealed no significant sex differences in urban vs rural distribution, nor differences in educational attainment by residence. Thus, the observed disparities in education and PROs are unlikely to be driven by residential factors alone. Additionally, the higher prevalence of single and widowed women highlights potential social support differences that could have impacted recovery and overall well-being after TAVI. Similarly, studies on patients undergoing cardiac catheterization showed that low educational attainment levels and unmarried status correlated with adverse outcomes.^12^^,^^13^

Furthermore, female patients experienced significantly higher anxiety levels both before and after the consent process. This finding aligns with previous studies indicating that women often report higher anxiety in medical settings.^14^ The elevated anxiety levels could affect their perception of the TAVI procedure and their overall hospital experience, highlighting the need for enhanced preprocedural counseling and support tailored to female patients. A recent meta-analysis provided compelling evidence that elevated anxiety levels were linked to poor prognoses and more complications in patients with acute myocardial infarction on a short-term and long-term scale.^15^

### Intraprocedural and postprocedural differences

Female patients were more likely to receive self-expandable prostheses, while male patients predominantly received balloon-expandable prostheses. This difference in prosthesis type is known from other TAVI studies and is mainly attributed to anatomical differences, such as smaller aortic annuli in women potentially affecting prosthetic type choices.^16^ Indeed, in this study, female patients showed on average a smaller aortic annular perimeter and area, possibly accounting for the use of supra-annular self-expandable devices in this patient cohort. Valve selection was based on individualized pre-procedural computed tomography planning and prosthesis sizing. Even though transvalvular pressure gradients are reportedly increased in balloon-expandable prostheses compared to self-expandable devices in patients with small annuli, a recent trial indicated no prognostic difference between balloon- and self-expandable prostheses types in these patients.^17^ Self-expanding valves might offer better hemodynamic performance with, however, a higher risk of paravalvular regurgitation and the need for pacemaker implantation.^12^^,^^14^ Despite similar complication rates in both sexes, women had a significantly longer hospital stay following TAVI. Prolonged hospitalization in females might be explained by their higher anxiety levels. Other studies have shown that different procedural approaches or other underlying health and social conditions, such as lack of social support, may require extended care.^18^ In the field of acute myocardial infarction, large-scale studies showed varying results of prolonged hospital LOS in female patients.^19^^,^^20^

At 90 days post-TAVI, female patients reported greater difficulties with daily activities, such as walking and shopping, as well as higher rates of depressive symptoms and feelings of helplessness compared to male patients. These data were confirmed in multivariable analyses. These outcomes suggest that female patients face more significant challenges in daily activities and mental health, which might be the consequence of aforementioned socioeconomic disparities and the cause for an increased risk of further adverse outcomes.^21^^,^^22^ Notably, PROs did not differ between the sexes when stratified by equivalent levels of educational attainment, suggesting that education may mitigate sex-based differences in perceived health status.

### Clinical implications

These observed sex differences in TAVI outcomes have 2 crucial implications for clinical practice: First, there is a need for personalized preprocedural education and psychological support to address the increased anxiety and lower educational levels among female patients. Enhanced educational tools may help bridge the comprehension gap and alleviate anxiety.^6^ Psychological support prior to the TAVI procedure could also mitigate anxiety. In addition to visual consent strategies, external resources such as the “CardioSmart” decision aid for aortic stenosis and TAVI may be valuable in promoting patient comprehension and shared decision-making, particularly among those with lower health literacy.^23^

Second, lack of social support and loneliness in elderly women may play a crucial role in impacting hospital LOS and PROs. Postprocedural patient pathways should therefore include strategies to provide social next to medical care, manage and mitigate psychological distress, and enhance functional recovery in women. Postprocedural interventions should furthermore focus on addressing physical disabilities, mental health issues, and potential isolation, which could potentially enhance patient satisfaction, functional recovery, and overall quality of life. To address the lack of social support reported more frequently by women in this cohort, a peer-based “TAVI buddy system” could be implemented, pairing patients scheduled for TAVI with individuals who have successfully undergone the procedure. This low-cost intervention may alleviate anxiety, promote empowerment, and facilitate recovery through shared experience.

### Study Limitations

This study has certain limitations. First, the sample size, although adequate for the analysis, may not fully capture the heterogeneity of the TAVI patient population. Second, the study is observational, and the findings might be influenced by unmeasured confounders. Further research with larger cohorts and randomized designs is warranted to validate these findings and explore the underlying mechanisms driving the observed sex differences, using one of the validated questionnaires assessing PROs that have been developed since the conduction of this trial.

## Conclusions

This analysis of the TAVI-COMIC randomized controlled trial highlights significant sex-specific differences in social and educational background, anxiety levels, procedural approaches, and postprocedural clinical and PROs in patients undergoing TAVI. Female patients encountered more significant difficulties in daily activities and mental health following the procedure, compounded by lower educational attainment and reduced social support. These findings underscore the need for sex-specific personalized care strategies to enhance the efficacy and satisfaction of TAVI treatment for both men and women. By addressing these differences, patient outcomes could be potentially optimized and the quality of life for TAVI patients further improved.

Perspectives **COMPETENCY IN MEDICAL KNOWLEDGE:** From a competency-based perspective, the findings underscore the importance of enhanced patient care and interpersonal and communication skills. Tailored preprocedural education, incorporating simplified consent processes and visual aids, can address sex disparities in comprehension and anxiety, improving patient engagement and shared decision-making. Additionally, integrating psychological and social support into systems-based practice is essential for addressing postprocedural challenges, particularly in female patients, who reported higher levels of depressive symptoms and reduced functional recovery.

**TRANSLATIONAL OUTLOOK:** Barriers to clinical application include the need for validated tools to assess PROs and strategies to personalize care based on sex-specific needs. Further research should focus on large-scale trials to confirm the observed disparities, exploring interventions that optimize education, psychological well-being, and social support. Multidisciplinary collaborations leveraging insights from psychosocial and cardiovascular domains could accelerate the integration of these findings into clinical practice, ultimately enhancing outcomes for diverse TAVI populations.

## Funding support and author disclosures

The study was supported by the DGK Center for Health Services Research (DGK-ZfKVF). The authors have reported that they have no relationships relevant to the contents of this paper to disclose.
