# Supplementary material for: Sex Differences in Clinical and Patient-Reported Outcomes in Transcatheter Aortic Valve Implantation: The TAVI-COMIC Trial
Source: JACC Adv. 2025 Jul 18;4(8):102012. doi: 10.1016/j.jacadv.2025.102012 (PMC12301741; doi:10.1016/j.jacadv.2025.102012)
Supplement: Supplemental_Material [file mmc1.pdf]

Supplemental Appendix

Supplemental Table 1. Patient-reported outcomes at 90 days following TAVI in female patients compared to male patients across different levels of educational attainment		
**Outcome**	**Odds ratio (95% CI) female vs. male**	**P value**
**Patients with a university degree (n=73)**		
Bad health status	0.34 (0.07-1.61)	0.16
General health status did not improve due to TAVI	0.72 (0.16-3.89)	0.68
15-minute walking disability	2.95 (0.62-13.50)	0.16
Helplessness	7.56 (1.36-47.71)	0.02
Depressive symptoms	2.33 (0.50-10.41)	0.27
Disability to go shopping	3.22 (0.75-14.26)	0.12
Dizziness	1.64 (0.38-7.59)	0.51
Syncope	NA	0.99
Dyspnoea (NYHA class II-IV)	1.0 (0.24-4.18)	1.00
Angina (CCS class I-IV)	1.50 (0.28-6.90)	0.61
**Patients with a high school degree (n=79)**		
Bad health status	0.51 (0.12-2.29)	0.36
General health status did not improve due to TAVI	0.50 (0.13-1.99)	0.31
15-minute walking disability	1.67 (0.38-6.72)	0.48
Helplessness	4.89 (0.92-28.92)	0.06
Depressive symptoms	1.50 (0.34-6.08)	0.58
Disability to go shopping	2.01 (0.50-7.74)	0.31
Dizziness	1.40 (0.37-5.32)	0.62
Syncope	NA	0.99
Dyspnoea (NYHA class II-IV)	0.91 (0.25-3.27)	0.88
Angina (CCS class I-IV)	1.20 (0.23-5.30)	0.82

Values are displayed as odds ratio and 95% CI, including P values. CCS, Canadian Cardiovascular Society; CI, Confidence Interval; NYHA, New York Heart Association; TAVI, transcatheter aortic valve implantation.

Supplemental Table 2. Association between socio-demographic parameters and post-procedural outcomes following TAVI between male and female patients

**Outcomes female vs. male**	**Body mass index**	**Age**	**High school degree**	**University degree**	**Employed**	**No relationship**	**STAI**
**Post-procedural complications and hospital data**							
Bleeding	1.01 (1.0–1.01)	0.99 (0.94–1.06)	1.34 (0.53–3.15)	1.66 (0.68–3.87)	1.58 (0.08–9.44)	1.48 (0.65–3.46)	1.06 (0.99–1.14)
Pneumonia	1.0 (0.99–1.0)	0.97 (0.91–1.03)	1.42 (0.52–3.57)	1.14 (0.39–2.92)	1.93 (0.10–11.62)	0.68 (0.26–1.66)	0.99 (0.91–1.07)
Bradycardia	1.0 (1.0–1.0)	1.01 (0.97–1.07)	1.01 (0.48–2.02)	1.03 (0.49–2.06)	0.80 (0.04–4.64)	1.09 (0.58–2.07)	1.01 (0.95–1.06)
Tachycardia	1.01 (0.99–1.02)	0.99 (0.86–1.19)	-	-	-	0.56 (0.03–5.92)	0.97 (0.78–1.17)
Cardiac conduction block	1.0 (0.99–1.0)	0.97 (0.93–1.02)	**0.39 (0.14–0.90)**	**0.40 (0.15–0.92)**	1.97 (0.28–8.88)	0.93 (0.49–1.77)	0.96 (0.90–1.01)
Stroke	1.0 (0.99–1.01)	0.97 (0.87–1.11)	0.69 (0.03–4.75)	0.70 (0.04–4.83)	-	1.71 (0.28–13.11)	1.09 (0.95–1.25)
TAVI unit LOS	1.0 (1.0–1.0)	1.0 (0.99–1.01)	0.92 (0.80–1.04)	0.90 (0.79–1.02)	1.17 (0.83–1.59)	1.05 (0.94–1.17)	**1.01 (1.0–1.02)**
Hospital LOS	1.0 (1.0–1.0)	1.01 (1.0–1.02)	0.94 (0.83–1.06)	0.95 (0.84–1.07)	1.13 (0.82–1.56)	1.06 (0.95–1.18)	**1.02 (1.01–1.02)**
**Patient-reported outcomes measures at 90 days after TAVI**							
Bad general health status	**1.01 (1.0–1.02)**	0.99 (0.92–1.05)	0.79 (0.36–1.79)	0.60 (0.28–1.31)	1.16 (0.18–22.61)	1.26 (0.61–2.65)	0.96 (0.90–1.03)
General health status did not improve due to TAVI	**1.01 (1.0–1.02)**	1.01 (0.96–1.06)	1.07 (0.53–2.23)	1.11 (0.55–2.31)	0.38 (0.07–2.13)	0.69 (0.37–1.28)	0.98 (0.92–1.03)
Helplessness	1.0 (0.99–1.01)	1.03 (0.97–1.09)	0.51 (0.20–1.18)	0.58 (0.23–1.30)	1.19 (0.06–9.55)	1.82 (0.91–3.68)	**1.11 (1.05–1.19)**
Depressive symptoms	1.0 (0.99–1.0)	1.01 (0.96–1.07)	0.53 (0.24–1.08)	0.56 (0.27–1.12)	1.86 (0.22–15.81)	**1.91 (1.05–3.52)**	**1.09 (1.03–1.15)**
Shopping disability	1.0 (0.99–1.01)	0.99 (0.95–1.05)	0.66 (0.32–1.32)	0.92 (0.46–1.79)	0.97 (0.13–5.10)	1.29 (0.71–2.35)	1.04 (0.98–1.01)
**Symptoms at 90 days after TAVI**							
Dizziness	0.99 (0.99–1.0)	1.02 (0.97–1.07)	0.85 (0.43–1.63)	0.95 (0.50–1.82)	6.8m (0.0–NA)	1.21 (0.68–2.14)	1.03 (0.98–1.09
Syncope	0.99 (0.97–1.01)	1.01 (0.91–1.14)	2.68 (0.64–10.59)	**6.70 (1.69–32.88)**	7.12 (0.33–63.36)	0.62 (0.13–2.41)	0.99 (0.87–1.11)
Dyspnoea	1.0 (0.99–1.01)	0.97 (0.92–1.02)	0.73 (0.38–1.42)	0.92 (0.48–1.76)	2.65 (0.33–54.11)	1.50 (0.83–2.72)	**1.06 (1.01–1.12)**
Angina	1.0 (0.99–1.01)	1.04 (0.98–1.11)	0.91 (0.39–1.99)	1.36 (0.62–2.90)	3.68 (0.43–31.48)	1.20 (0.59–2.45)	**1.07(1.0–1.14)**

The risk of each outcome was compared between female patients to male patients. Results are displayed and odds ratio or incidence rate ratio and 95% confidence interval. STAI, State-Trait Anxiety Inventory; TAVI, transcatheter aortic valve implantation.
